# Effects of radiotherapy and estramustine on the microvasculature in malignant glioma

**DOI:** 10.1038/sj.bjc.6690333

**Published:** 1999-04

**Authors:** M Johansson, A T Bergenheim, A Widmark, R Henriksson

**Affiliations:** Departments of Oncology, andNeurosurgery, Umeå University, SE 901 85 Umeå, Sweden

**Keywords:** angiogenesis, chemotherapy, glioma, radiotherapy, VEGF

## Abstract

Tumour angiogenesis is essential for progression of solid tumours and constitutes an interesting target for therapy. However, impaired tumour blood supply may also be an important obstacle for treatment by radiotherapy and chemotherapy. Estramustine has been shown to increase tumour blood flow and potentiate the effect of radiotherapy in experimental glioma. This study investigated the effects of fractionated radiotherapy and estramustine on angiogenesis in malignant glioma. The intracerebral BT4C rat glioma model was used and the animals were given whole brain radiotherapy 4 Gy × 5 days alone or in combination with estramustine 20 mg kg^-1^ i.p. daily. Tumour microvascular density (MVD) was assessed by manual and computerized morphometrical analysis. Expression of vascular endothelial growth factor (VEGF) was studied by in situ hybridization. Radiotherapy decreased MVD to 157 vessels per mm^2^ compared to 217 vessels per mm^2^ in controls. Estramustine counteracted this anti-angiogenic effect and potentiated the anti-tumoural effect of radiotherapy. In addition, vessel size increased after estramustine treatment. Five days after completion of radiotherapy the expression of VEGF was increased in the centre of the tumours. In conclusion, fractionated radiotherapy decreases microvascular density in experimental malignant glioma. This effect was abolished by estramustine. The anti-vascular effect of irradiation is important to recognize when combining radiotherapy with cytotoxic drugs. © 1999 Cancer Research Campaign


*British Journal of Cancer* (1999) **80**(1/2), 142–148
© 1999 Cancer Research Campaign
Article no. bjoc.1998.0333

### Effects of radiotherapy and estramustine on the microvasculature in malignant glioma

**M Johansson[1], AT Bergenheim[2], A Widmark[1] and R Henriksson[1]**

Departments of [1]Oncology and [2]Neurosurgery, Umeå University, SE 901 85 Umeå, Sweden

**Summary** Tumour angiogenesis is essential for progression of solid tumours and constitutes an interesting target for therapy. However, impaired tumour blood supply may also be an important obstacle for treatment by radiotherapy and chemotherapy. Estramustine has been shown to increase tumour blood flow and potentiate the effect of radiotherapy in experimental glioma. This study investigated the effects of fractionated radiotherapy and estramustine on angiogenesis in malignant glioma. The intracerebral BT4C rat glioma model was used and the animals were given whole brain radiotherapy 4 Gy × 5 days alone or in combination with estramustine 20 mg $kg^{-1}$ i.p. daily. Tumour microvascular density (MVD) was assessed by manual and computerized morphometrical analysis. Expression of vascular endothelial growth factor (VEGF) was studied by in situ hybridization. Radiotherapy decreased MVD to 157 vessels per $mm^2$ compared to 217 vessels per $mm^2$ in controls. Estramustine counteracted this anti-angiogenic effect and potentiated the anti-tumoural effect of radiotherapy. In addition, vessel size increased after estramustine treatment. Five days after completion of radiotherapy the expression of VEGF was increased in the centre of the tumours. In conclusion, fractionated radiotherapy decreases microvascular density in experimental malignant glioma. This effect was abolished by estramustine. The anti-vascular effect of irradiation is important to recognize when combining radiotherapy with cytotoxic drugs.

**Keywords:** angiogenesis; chemotherapy; glioma; radiotherapy; VEGF

An increasing amount of evidence supports the hypothesis that tumours are dependent on a sufficient blood supply for their maintenance and growth (Folkman, 1971, 1990). Its clinical importance has been demonstrated by the finding of tumoural neovascularization as a negative prognostic factor in a number of tumours (Weidner, 1993; Gasparini and Harris, 1995). Hypoperfusion of tumours with resulting hypoxia is considered to be one of the major causes of radiotherapy failure (Gray, 1961; Littbrand and Révész, 1969; Hall, 1994). Systemically administered cytotoxic agents are obviously dependent on the vasculature of the tumour to reach their target. An impaired tumour vasculature may limit the amount of drug reaching the blood–tumour interface (Jain, 1987). In this sense, a high grade of tumour vascularization facilitates the effects of treatment and, at the same time, paradoxically it implies a poor prognosis.

Malignant glioma, the most common malignant brain tumour, is a highly vascularized tumour in which a high grade of neovascularization has been shown to be a negative prognostic factor (Leon et al, 1996). The prognosis, in spite of surgical resection and radiotherapy, is poor due to its diffusely infiltrative growth pattern (Kelly et al, 1987). Estramustine phosphate is a cytotoxic agent with demonstrated anti-tumoural effects in experimental glioma models in vitro as well as in vivo (Bergenheim et al, 1994*c*; von Schoultz et al, 1988). Its metabolite, estramustine (EaM), has been demonstrated to enhance the effect of radiotherapy (Bergenheim et al, 1995), which may be explained by the induced arrest of tumour cells in G2/M phase of the cell cycle (Yoshida et al, 1994; Bergenheim et al, 1995) as well as to the demonstrated increase in glioma blood flow (Johansson et al, 1997).

*Received 3 June 1998*
*Revised 6 August 1998*
*Accepted 14 August 1998*

*Correspondence to:* M Johansson

In this study, we have evaluated the effects of radiotherapy and EaM on vascularization in malignant glioma using an experimental rat model. We have also studied the effect on the expression of mRNA for vascular endothelial growth factor (VEGF) as one of the most important stimulating factors for neovascularization (Ferrara, 1996).

### MATERIALS AND METHODS

### Tumour model

The previously characterized syngenic intracerebral BT4C rat glioma model was used for this study (Bergenheim et al, 1994*a*). Prior to implantation, tumour cells (originally a generous gift from Prof. Bjerkvig, Bergen, Norway) were suspended in minimal essential medium (MEM, Flow laboratories, Glasgow, UK) and supplemented with 5% rat serum. BT4C rat glioma cells were thereafter transplanted intracerebrally using a stereotactic frame (Kopf 900, David Kopf, Tujunga, CA, USA). A total of 20 000 tumour cells in a volume of 5 μl were deposited in the caudate nucleus, 3.5 mm right of bregma and at a depth of 4.5 mm, using a microsyringe (22G, Unimetrics, Shorewood, IL) fitted to the micromanipulator of the stereotactic frame. Animals were housed under controlled conditions and fed ad libitum. The experiments were approved by the local ethics committee for animal research.

### Treatment schedule

A total of 26 rats were divided into four groups after implantation. Two groups received radiotherapy ($n$ = 7) or EaM ($n$ = 6) alone and one group received a combination of radiotherapy and EaM ($n$ = 6). The fourth group served as untreated controls ($n$ = 7). Since EaM is the most active metabolite to accumulate in glioma tissue, EaM was used for this study (Bergenheim et al, 1993,

1994*b*; Johansson et al, 1998). EaM was dissolved in ethanol and castor oil (2:8) giving a concentration of 10 mg $ml^{-1}$. EaM treatment started on day 7 after tumour implantation and was given as daily 20 mg $kg^{-1}$ intraperitoneal (i.p.) injections until sacrifice on day 24. The dose of EaM given was obtained from previous studies showing that 20 mg $kg^{-1}$ EaM daily exerts a significant anti-tumoural action with a low grade of toxicity (Bergenheim et al, 1994*a*). Day 24 was chosen for sacrifice since the animals do not suffer from severe neurological symptoms at this time point and the untreated tumour size is known to be representative for the final outcome (Bergenheim et al, 1994*a*).

Radiotherapy was given as fractionated whole brain irradiation using a conventional 4 MV linear accelerator. Treatment was given as daily 4 Gy fractions for 5 consecutive days starting on day 12 after tumour implantation. Source surface distance was 0.66 m and the dose rate 2 Gy $min^{-1}$. Irradiation was performed on conscious rats temporarily immobilized in a net restrainer. The dose and fractionation was chosen according to previous experience with the purpose to obtain a moderate tumour effect without inflicting serious normal brain tissue damage (Henderson et al, 1981; Bergenheim et al, 1995).

### Tissue preparation and tumour size assessment

After sacrifice, brains were immediately removed and fixed overnight in phosphate-buffered 4% formaldehyde solution. Brains were thereafter kept cold in 70% ethanol until further analysis was performed. Tumour size was evaluated as volume. Height and width was measured at the largest coronal section using a calliper. Tumour volume was thereafter calculated using the formula for the ellipsoid ($r1 \times r2 \times r3 \times \pi \times 4/3$) where the radius in the sagittal plane was approximated to the same as the coronal radius. The rat brains were than dehydrated in graded ethanol series and embedded in paraffin. For morphological studies, including in situ hybridization, 4-μm sections were cut using a sledge microtome. Tissue sections were mounted on polylysin-covered microscope slides for immunohistochemistry and on Super Frost/Plus slides (Histolab, Västra Frölunda, Sweden) for in situ hybridization.

### Vascular staining and microvascular density

Vessels in the BT4C brain tumours were immunohistochemically stained for factor VIII and quantified manually using a method originally presented by Weidner (1993). Sections chosen for immunohistochemical staining were deparaffinized in xylene, dehydrated in graded ethanol series and washed in deionized water. The tissue was then permeabilized in 0.05% protease P27 at 37°C for 30 min and the digestion was stopped by immersion in 95% ethanol for 5 min followed by washing in phosphate-buffered saline (PBS) for 5 min × 4. Slides were then incubated with normal goat serum for 20 min prior to 60-min incubation with rabbit anti-rat factor VIII antibody (DAKO A/S, Glostrup, Denmark) diluted 1:200. After washing in PBS 5 min × 4 slides were incubated with a biotinylated goat anti-rabbit antibody (Vector laboratories, Burlingame, CA) and washed in PBS 5 min × 4 again. Sections were subsequently incubated with avidin linked alkaline phosphatase (Vector ABC-AP) for 20 min according to the vendors recommendations. Development was finally performed using alkaline phosphatase reagent (Vector) diluted in Tris buffer. Endogenous alkaline phosphatase activity was blocked by adding 1 mM Levamisole to the reagent buffer. After 5 min washing in tap water, slides were briefly immersed in 95% ethanol and mounted in gelatine glycerol.

Assessment of microvascular density (MVD) was performed by manual counting in selected areas with high vascular density (hot spots). Each tumour was scanned at low magnification and four hot spots were chosen for MVD quantification. All stained objects within a 200× field (area 1.227 $mm^2$) were counted using a standard light microscope (Axiophot, Zeiss, Oberkochen, Germany). Each hot spot was counted twice and the arithmetical mean in each spot was used to calculate the mean MVD for each tumour section which was used for further analysis.

In order to obtain quantitative information on tumour vessel size, and to validate the manual counting of vessels, the sections were also analysed using a computerized image analysis system (CIAS). The system used for obtaining images consists of a standard microscope (Axiophot, Zeiss, Oberkochen, Germany) fitted with a high resolution digital camera (ProgRes 3000, Kontron Elektronik GmbH, Eching bei München, Germany) under control of WinCam 1.4 software (CCD-Videometrie, Unterscheissheim, Germany) on a PC computer. Images were then analysed using KS-400 2.08 software (Kontron Elektronik). The image analysis procedure was automated by a KS-400 macro with an individual threshold setting for each section. Threshold setting was subjectively verified by superimposing the contours of the obtained binary image on the original. If detectable mismatch was found, the procedure of thresholding was redone until matching was acceptable. Four vascular hotspots were identified by the same procedure as for manual counting and the CIAS was used to obtain data from each spot at 200× magnification (area 0.307 $mm^2$). For each vessel in each area, the perimeter, area, maximal diameter and minimal diameter were measured and stored in a database. Means for each tumour were calculated and used for further statistical analysis of treatment groups. Frequency distributions of the vessel areas were based on the entire database of each treatment group.

### RT-PCR and in situ hybridization

The mRNA expression of VEGF was evaluated by in situ hybridization in histological sections from four different tumours in each treatment group and in ten controls. In situ hybridization for VEGF was performed mainly as previously described (Lindgren et al, 1997); however, it was modified for formalin-fixed paraffin-embedded sections. RNA was isolated from a BT4C tumour using TRIzol reagent (GIBCO BRL, Life Technologies, Stockholm, Sweden) according to the instructions of the manufacturer. Specific polymerase chain reaction (PCR) primers were selected for rat glioma-derived VEGF. Reverse transcriptase PCR (RT-PCR) was thereafter performed on 1 μg tumour RNA using a One Strand cDNA synthesis kit (Boehringer Mannheim, Stockholm, Sweden) and the reverse primer for VEGF. The cDNA synthesis was followed by a standard PCR protocol where annealing temperature was set to 45°C. This resulted in a PCR product of 404 bp. The fragments were cloned into a plasmid carrying T3 and T7 DNA polymerase promotors on opposite sides of the cloning cassette (Bluescript SK +/–, Stratagene, La Jolla, CA, USA). The VEGF fragment was inserted in the SmaI site of the cloning cassette. Orientation of fragment was confirmed by restriction site analysis.

A digoxin (DIG)-labelled RNA probe was obtained by using a DIG–RNA kit (Boehringer Mannheim, Stockholm, Sweden) and plasmids linearized with appropriate restriction enzymes. Probes

© Cancer Research Campaign 1999 

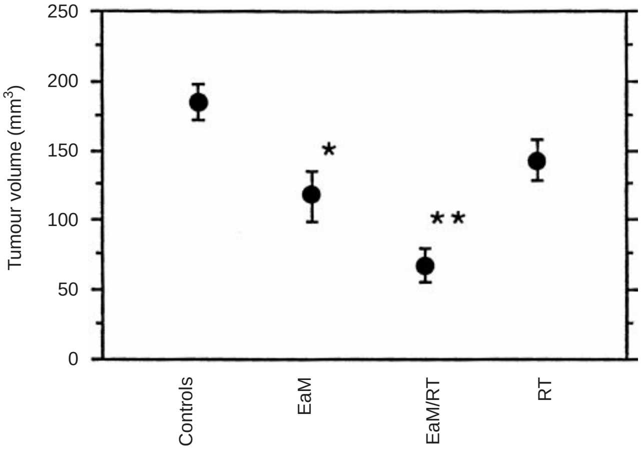

**Figure 1** Brain tumour volume (mm³) in control animals and animals treated with estramustine (EaM), radiotherapy alone (RT) or radiotherapy combined with estramustine (EaM/RT). Values are given as means ± s.e.m. $^*P < 0.05$, $^{**}P < 0.01$ compared to control

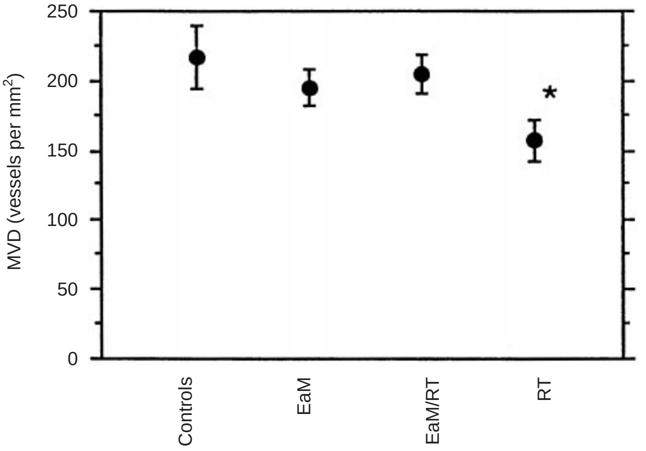

**Figure 2** MVD in intracerebral glioma expressed as vessels per mm² in control animals and animals treated with estramustine (EaM), radiotherapy alone (RT) or radiotherapy combined with estramustine (EaM/RT). Values are given as means ± s.e.m. $^*P < 0.05$ compared to control

**Table 1** Computerized image analysis of microvascular density (MVD), perimeter, area, minimum diameter and maximum diameter in treated and not treated intracerebral rat glioma. Values given as mean ± SE

| Group | MVD (vessels/mm²) | Perimeter (μm) | Area (μm²) | Min diameter (μm) | Max diameter (μm) |
|---|---|---|---|---|---|
| Controls | 250 ± 25 | 89 ± 8 | 145 ± 20 | 11 ± 0.7 | 22 ± 1.3 |
| EaM | 203 ± 11 | 129 ± 7** | 352 ± 36** | 15 ± 0.6** | 32 ± 1.3** |
| EaM/RT | 200 ± 14 | 124 ± 7* | 278 ± 15** | 14 ± 0.4* | 31 ± 0.9** |
| RT | 179 ± 12* | 82 ± 4 | 115 ± 11 | 10 ± 0.4 | 21 ± 0.9 |

$^*P < 0.05$, $^{**}P < 0.01$ compared to control.

were manufactured according to the vendors' instructions and purified on NucTrap purification columns (Stratagene, La Jolla, CA, USA).

After dewaxing in fresh xylene for at least 60 min, sections were rehydrated through a graded ethanol series. Sections were then fixed in 4% paraformaldehyde/77 mM $Na_2HPO_4$, 23 mM $NaH_2PO_4$, 0.15 M NaCl (PBS) for 10 min and washed in PBS 5 min × 2. To facilitate probe penetration, sections were incubated in 0.2 M HCl, washed in PBS 5 min × 2, acetylated for 10 min in a buffer containing 1.33% vv triethanolamine, 0.175% vv HCl and 0.25% acetic anhydride followed by washing in PBS 5 min × 2. To further break formalin induced protein changes, sections were incubated at 37°C with proteinase K 25 μg $ml^{-1}$ for 30 min and washed in PBS 5 min × 2. Slides were then prehybridized in 25% formamide, 5 × saline–sodium citrate (SSC), 5 × Denhardt (Sigma, Stockholm, Sweden), 250 μg $ml^{-1}$ Bakers yeast RNA and 500 mg $ml^{-1}$ salmon sperm DNA in 4°C overnight or 10 min at RT in a chamber humidified with 50% formamide and 5 × SSC. Hybridization was performed with DIG-labelled RNA probes at a concentration of 50–200 ng 100 $\mu l^{-1}$ hybridization buffer, similar to the prehybridization buffer but supplemented with 10% dextransulphate. Hybridization was carried out at 70°C overnight and sections were protected from drying with siliconized glass coverslips (Sigmacoat, Sigma). All incubations to this step were carried out in RNAse-free containers and all buffers were diluted in di-ethylpyrocarbonate-treated deionized water. Following hybridization, coverslips were removed in 70°C 5 × SSC and sections were then equilibrated in 0.2 × SSC, 0.1 M Tris pH 7.5, 0.15 M sodium chloride (NaCl) (B1) for 60 min. Sections were then incubated in 10% fetal calf serum (FCS)/B1 for at least 60 min. The DIG-labelled probes were tracked by incubating sections with anti-DIG fabs (Boehringer Mannheim, Stockholm, Sweden), diluted 1:5000 in 1% FCS/B1, at 4°C overnight. Development was carried out at 30°C with 75 mg $ml^{-1}$ 5-bromo-4-chloro-3-inolyl-phosphate (Boehringer Mannheim, Stockholm, Sweden), 50 mg $ml^{-1}$ 4-nitro blue tetrazoliumchloride (Boeringer Mannheim, Stockholm, Sweden) in a buffer consisting of 10% polyvinyl alcohol in 0.1 M Tris pH 9.0, 0.1 M NaCl and 5 mM magnesium chloride. Development was stopped after 12 h by immersing slides in deionized water. Finally, sections were mounted in gelatine glycerol medium.

The VEGF expression was semiquantitatively evaluated by two of the authors on blinded sections. The staining was expressed as missing, weak, moderate or pronounced.

### Statistical analysis

Values are expressed as means ± s.e.m. For comparisons between groups, the Mann–Whitney *U*-test was used. A *P*-value less than 0.05 was considered to be statistically significant. Linear regression was used for correlation of manual and CIAS-aided MVD assessment. Statistical analysis was performed using the StatView 4.5 software (Abacus Concepts, Berkley, CA, USA) for the Power Macintosh (Apple Computer, Cupertino, CA, USA) computer.

 © Cancer Research Campaign 1999

### RESULTS

### Tumour size

The combination of EaM and radiotherapy decreased tumour volume significantly and was the most effective treatment administered in this experiment when compared to controls (Figure 1). EaM/RT treatment reduced the tumour volume to 69 ± 12 $mm^3$ compared to a volume of 185 ± 13 $mm^3$ in the control group ($P < 0.01$). EaM alone also decreased tumour volume but to a lower degree than the combination treatment (117 ± 18 $mm^3$; $P < 0.05$). Radiotherapy alone caused a small decrease in tumour volume (143 ± 15), which was not significant in this experiment.

### Microvascular density

When MVD was manually assessed, radiotherapy alone significantly decreased mean MVD to 157 ± 14 vessels per $mm^2$ compared to controls where mean MVD was found to be 217 ± 22 vessels per $mm^2$ ($P < 0.05$) (Figure 2). EaM, when given alone, did not affect tumour MVD (195 ± 12 vessels per $mm^2$) and, interestingly, when EaM was given in combination with RT no reduction in tumour MVD could be observed (205 ± 13 vessels per $mm^2$).

MVD assessed by computerized image analysis showed reasonable correlation to the manually obtained data ($r = 0.65$) and radiotherapy was also found to decrease MVD ($P < 0.05$) when analysed with CIAS (Table 1).

### Microvessel size

Individual tumour vessel size increased significantly when EaM was administered (Table 1). Mean vessel area, perimeter and diameter all increased significantly in the EaM and EaM/RT groups. The distribution of individual vessel area for the different treatment groups is shown in frequency distribution histograms in Figure 3.

### VEGF in situ hybridization

A high expression of VEGF mRNA was found in the invasive tumour border in all groups (Figure 4A) while the expression was mostly absent in the centre of the tumours (Figure 4B). However, 5 days after completion of radiotherapy we found an increased cellular expression of VEGF scattered in the tumour centre (Table 2).

### DISCUSSION

This study demonstrated that the tumoural vascular network is significantly affected by fractionated irradiation with a decrease in microvessel density. EaM seems to abolish this effect concomitant with an enhanced anti-tumoural effect. Since tumour vascularization is of utmost importance for tumour progression, it is suggested that the anti-vascular effect of irradiation may be partly involved in the anti-tumoural effect of radiotherapy. On the other hand, an impaired tumoural vascular network may reduce oxygenation and thus give a negative effect on tumour cell damage. Therefore, the vascular effect demonstrated may be a radiobiological Janus face where decreased oxygenation may outnumber the possible positive treatment effects on tumour growth through the vascular damage. In this sense the effect of EaM can be of therapeutic interest.

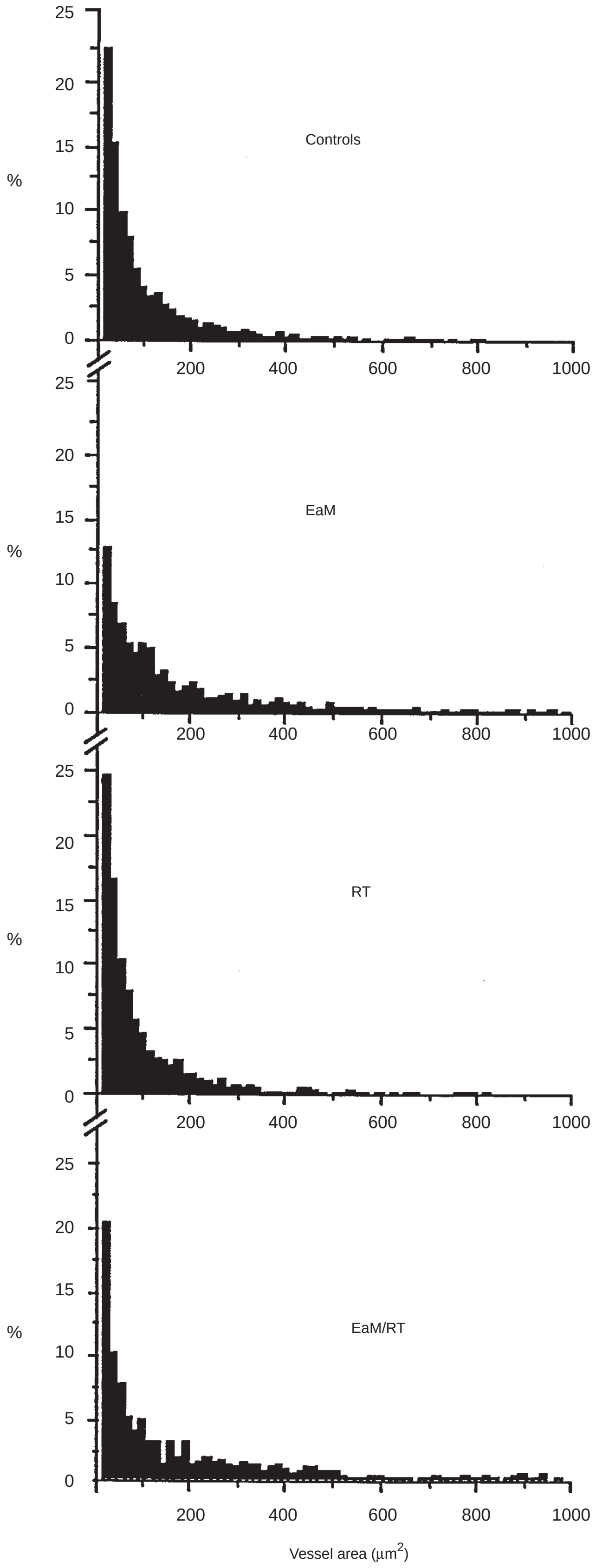

**Figure 3** Frequency distribution of vessel area ($\mu m^2$) in intracerebral glioma in control animals and animals treated with estramustine (EaM), radiotherapy alone (RT) or radiotherapy combined with estramustine (EaM/RT)

© Cancer Research Campaign 1999

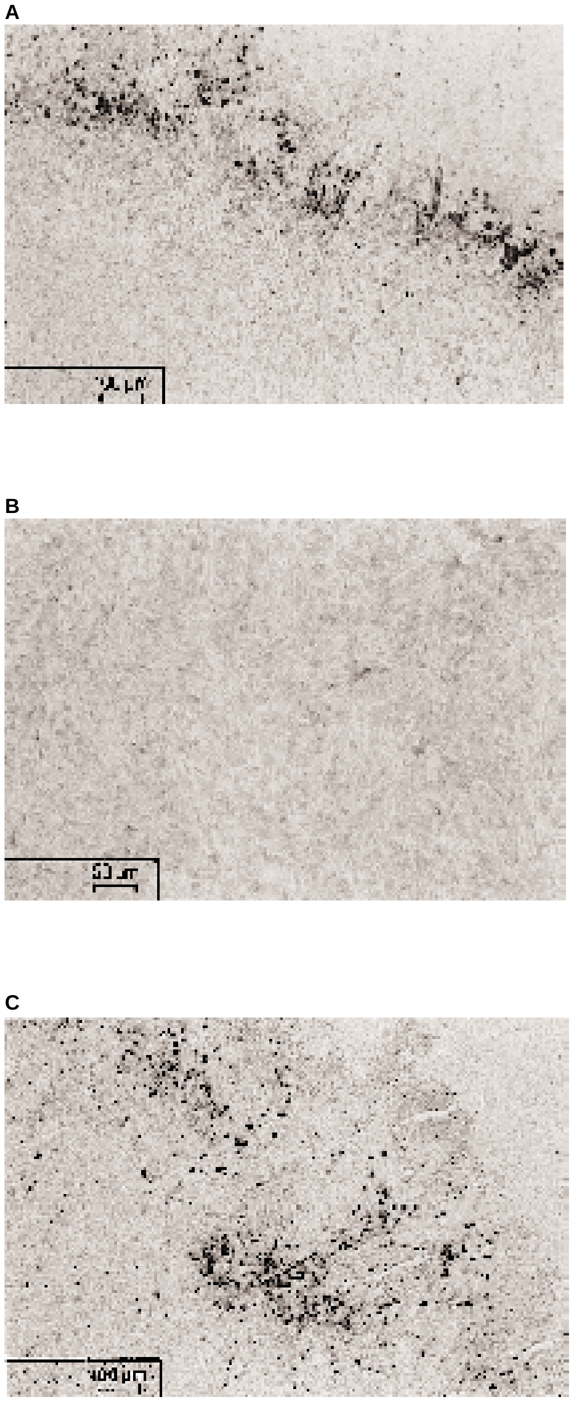

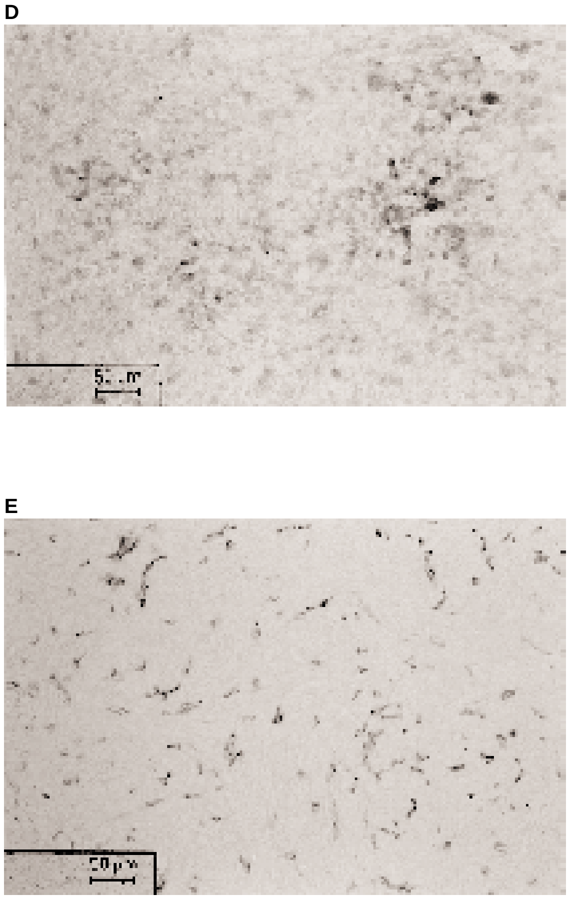

**Figure 4** VEGF in situ hybridization demonstrating the high expression in tumour border in control animals (**A**) and no expression in tumour centre (**B**) of the same control tumour. Also in the radiotherapy group, a high VEGF expression in the tumour border is seen (**C**) but, in the tumour centre of irradiated tumours, clusters of VEGF-expressing cells indicate a picture different from controls (D). Factor VIII immunohistochemistry of a vascular hotspot in a control animal where mean microvascular density is 252 vessels per mm$^2$ (**E**)

The effect of radiotherapy on normal tissue vasculature is well known and believed to be the main cause of late radiation damage (Baker and Krochak, 1989). The slow turnover rate of non-proliferating endothelial cells leads to delayed damage to the vasculature. However, the effect on tumour vasculature may be different. The rapid turnover rate of proliferating endothelial cells in the tumour stroma (Denekamp and Hobson, 1982) make them potentially more vulnerable to irradiation than normal tissue and, therefore, it has been hypothesized that tumour stroma may be an important target for radiotherapy (Denekamp, 1991). Our study supports this hypothesis. Since no acute effects on the expression of VEGF in tumour tissue during irradiation could be demonstrated, the anti-vascular effect may well be mediated by direct antiproliferative effects on the endothelial cells. The elevated expression of VEGF in the central tumour demonstrated 5 days after radiotherapy may be induced by an increased oxygen demand in the tissue where angiogenesis was hampered by irradiation. Hypoxia is known to be the most important physiological stimuli for VEGF up-regulation in vitro (Shweiki et al, 1992) as well as in vivo (Plate et al, 1992, 1993).

*© Cancer Research Campaign 1999*

**Table 2** The expression of VEGF detected by in situ hybridization in the border and centre of malignant rat glioma. Staining quantified in a four-graded scale where 0 = no staining; 1 = weak staining; 2 = moderate and 3 = intensive staining

| | Staining intensity | | | | | | | |
|---|---|---|---|---|---|---|---|---|
| | Tumour border | | | | Tumour centre | | | |
| | 0 | 1 | 2 | 3 | 0 | 1 | 2 | 3 |
| Controls (*n* = 5) | 0 | 1 | 1 | 3 | 2 | 3 | 0 | 0 |
| EaM (*n* = 4) | 0 | 0 | 3 | 1 | 0 | 4 | 0 | 0 |
| EaM/RT (*n* = 4) | 0 | 1 | 3 | 0 | 1 | 3 | 0 | 0 |
| RT (*n* = 4) | 0 | 0 | 3 | 1 | 0 | 2 | 1 | 1 |

The effect of radiotherapy on tumour angiogenesis, however, has been less extensively studied. In tumour tissue most experimental studies have focused on the functional vasculature. A decrease of functional tumour vessels, days to weeks after single-dose irradiation, has been reported in a mouse mamary carcinoma model (Hilmas and Gillette, 1975) as well as in a human melanoma xenograft (Solesvik et al, 1984). In other studies, an initial increase in functional tumour vessels followed by decrease was reported in a mouse neuroblastoma after single-dose irradiation (Song et al, 1974) and in a rat rhabdomyosarcoma during fractionated radiotherapy (Zywietz, 1990). The increase of functional tumour vessels shortly after irradiation has also been reported (Rubin and Casarett, 1966; Dewhirst et al, 1990). The supervascularized state in at least some regressing tumours is believed to be due to a relative increase of the tumour stromal compartment rather than increased tumour angiogenesis. In models of experimental angiogenesis, single-dose radiotherapy has been shown to inhibit in vivo angiogenesis in the chick embryo chorioallantoic membrane (CAM) assay (Hatjikondi et al, 1996) as well as in the disc angiogenesis model in the rat (Prionas et al, 1990).

Most studies, although using other techniques, are in concordance with our results and further validate the finding that irradiation may inhibit tumour angiogenesis. To our knowledge, the effect of fractionated radiotherapy on the MVD in experimental tumours has not previously been described quantitatively using an endothelium-specific marker. Interestingly, a limited clinical study indicates that preoperative radiotherapy may decrease intratumoural vascular density in patients with malignant glioma (Seiler et al, 1979).

EaM has previously been shown to have radiosensitizing effects in vitro (Yoshida et al, 1994; Bergenheim et al, 1995) as well as in vivo (Bergenheim et al, 1995). The mechanism has been coupled to the ability of EaM to block tumour cells in the vulnerable G2/M phase of the cell cycle (von Schoultz et al, 1988). The radiosensitizing effect of EaM is more pronounced in vivo than in vitro, an observation that suggests that other mechanisms, such as vascular effects, are of importance. Earlier, we showed that EaM increases tumour blood flow (TBF) in the BT4C rat glioma model (Johansson et al, 1997). In this study EaM reduced the anti-angiogenic effect of radiotherapy and potentiated the treatment effects as previously shown. The increase in TBF, together with the vaso-radioprotective effects seen in this study, may be a physiological mechanism by which EaM acts as a radiosensitizer. It is well-established that tumour hypoxia is an important cause of radioresistance (Hall, 1994) and EaM may act as a radiosensitizer through enhancement of tumour oxygenation.

The mechanism by which EaM reduces the negative effects of irradiation on tumour angiogenesis is not known. Estradiol, one of the components of the EaM conjugate, is known to promote proliferation and migration of human umbilical vein cells in vitro (Morales et al, 1995). Estradiol has also been shown to up-regulate VEGF mRNA in normal endometrium (Cullinan Bove and Koos, 1993) as well as in a chemically induced rat mammary carcinoma (Nakamura et al, 1996). An increase of VEGF protein, as well as increased MVD and tumour vessel size 7 days after estradiol treatment, has been reported in an estradiol-induced rat pituitary tumour model (Banerjee et al, 1997). One mechanism by which estradiol may act on tumour vasculature could, therefore, be the induction of the VEGF system. In our study we could not confirm any increase in VEGF expression after EaM treatment. However, based on this study, altered expression of VEGF or any other angiogenic factor cannot be excluded as the mechanism by which EaM protects irradiated tumour vasculature.

Although in a totally different tumour model, our findings that an estradiol-based cytotoxic conjugate induces increased TBF and increased mean vessel size suggests that similar oestrogen-related mechanisms to those in the pituitary model may be involved. This is supported by the fact that estradiol alone also increases TBF in the BT4C rat glioma model (Johansson et al, 1997). The increased mean vessel size after EaM treatment seen in this experiment may be due to an induction of tumour vessel growth or a tumoural vasodilatation which could explain the previously reported increase in TBF. The levels of free estradiol in tumour tissue after EaM treatment are low but may be sufficient to induce growth and dilatation or enlargement of tumour microvasculature in irradiated tumours (Johansson et al, 1998). In the clinical situation, estradiol levels are at least high enough to induce the oestrogenic side-effects recognized after treatment with estramustine phosphate (Bergenheim et al, 1996). Another explanation could be that the intact EaM conjugate exerts estradiol-like actions when it comes to modulating TBF and neovascularization. Somewhat puzzling, however, EaM did not increase tumour MVD in non-irradiated tumours. Maybe the vascular density in these tumours is so high that the angiogenesis cascades are saturated, and only when angiogenesis is suppressed, is there space for increased tumour MVD.

In the present study, quantification of vascularization was performed by manual counting of vessels and aided by a CIAS. The correlation between the methods was found to be acceptable and both methods displayed the same differences between treatment groups. There are two possible explanations for the absolute differences in MVD counts (Figure 2 and Table 1) are two. First, perfect correlation cannot be expected since the two measurements were not performed on the very same subjectively chosen vascular hot spots. Second, due to technical limitations (camera CCD element size), the area analysed using the CIAS was only one-quarter of the

© Cancer Research Campaign 1999

area counted manually. This is believed to increase measurement scatter since the optimal counting area is larger. Taking this into consideration, we believe that the correlation between the methods is good and further validates the findings reported. In this study, computerized colour image analysis was used as a complement to manual counting, but the results reported encourage further refinement of the method. Moreover, the CIAS proved powerful in providing individual vessel size data.

To conclude, this study demonstrates that fractionated radiotherapy inhibits tumour angiogenesis in a rat glioma model. This may be a part of the treatment effect of radiotherapy. However, the decrease in vascularization could make drug delivery to tumours even more difficult post-irradiation. Interestingly, the estradiol-linked cytotoxic agent, EaM, concomitantly counteracted the anti-angiogenic effects of radiotherapy and increased the irradiation-induced growth inhibition. Therefore, this study suggests that the treatment schedule is important when combining radiotherapy with other treatment modalities such as chemotherapy.

### ACKNOWLEDGEMENTS

This study was supported by grants from the Swedish Society Against Cancer (RMC) and the Lion's Cancer Research Foundation, Umeå University, Sweden. Kerstin Berg, Britt-Inger Gladzki, Elisabeth Karlsson and Charlotte Nordström are acknowledged for their skillful technical assistance. Per-Olov Löfroth is acknowledged for his unvaluable help with animal dosimetry.

### REFERENCES

Baker DG and Krochak RJ (1989) The response of the microvascular system to radiation: a review. *Cancer Invest* **7**: 287–294

Banerjee SK, Sarkar DK, Weston AP, De A and Campbell DR (1997) Overexpression of vascular endothelial growth factor and its receptor during the development of estrogen-induced rat pituitary tumors may mediate estrogen-initiated tumor angiogenesis. *Carcinogenesis* **18**: 1155–1161

Bergenheim AT, Gunnarsson PO, Edman K, von Schoultz E, Hariz MI and Henriksson R (1993) Uptake and retention of estramustine and the presence of estramustine binding protein in malignant brain tumours in humans. *Br J Cancer* **67**: 358–361

Bergenheim AT, Elfversson J, Gunnarsson P-O, Edman K, Hartman M and Henriksson R (1994*a*) Cytotoxic effect and uptake of estramustine in a rat glioma model. *Int J Oncol* **5**: 293–299

Bergenheim AT, Björk P, Bergh J, von Schoultz E, Svedberg H and Henriksson R (1994*b*) Estramustine-binding protein and specific binding of the anti-mitotic compound estramustine in astrocytoma. *Cancer Res* **54**: 4974–4979

Bergenheim AT, Zackrisson B, Elfverson J, Roos G and Henriksson R (1995) Radiosensitizing effect of estramustine in malignant glioma in vitro and in vivo. *J Neuro-Oncol* **23**: 191–200

Bergenheim AT, Henriksson R, Piepmeier JM and Yoshida D (1996) Estramustine in malignant glioma. *J Neuro Oncol* **30**: 81–89

Cullinan Bove K and Koss RD (1993) Vascular endothelial growth factor/vascular permeability factor expression in the rat uterus: rapid stimulation by estrogen correlates with estrogen-induced increases in uterine capillary permeability and growth. *Endocrinology* **133**: 829–837

Denekamp J (1991) The current status of targeting tumour vasculature as a means of cancer therapy: an overview. *Int J Radiat Biol* **60**: 401–408

Denekamp J and Hobson B (1982) Endothelial-cell proliferation in experimental tumours. *Br J Cancer* **46**: 711–720

Dewhirst MW, Oliver R, Tso CY, Gustafson C, Secomb T and Gross JF (1990) Heterogeneity in tumor microvascular response to radiation. *Int J Radiat Oncol Biol Phys* **18**: 559–568

Ferrara N (1996) Vascular endothelial growth factor. *Eur J Cancer* **32a**: 2413–2422

Folkman J (1971) Tumor angiogenesis: therapeutic implications. *N Engl J Med* **285**: 1182–1186

Folkman J (1990) What is the evidence that tumors are angiogenesis dependent? (editorial). *J Natl Cancer Inst* **82**: 4–6

Gasparini G and Harris AL (1995) Clinical importance of the determination of tumor angiogenesis in breast carcinoma: much more than a new prognostic tool. *J Clin Oncol* **13**: 765–782

Gray LH (1961) Radiobiologic basis of oxygen as a modifying factor in radiation therapy. *Am J Roentgenol* **85**: 803

Hall EJ (1994) *Radiobiology for the Radiologist*. Lippincott: Philadelphia

Hatjikondi O, Ravazoula P, Kardamakis D, Dimopoulos J and Papaioannou S (1996) In vivo experimental evidence that the nitric oxide pathway is involved in the X-ray-induced antiangiogenicity. *Br J Cancer* **74**: 1916–1923

Henderson SD, Kimler BF and Morantz RA (1981) Radiation therapy of 9L rat brain tumors. *Inl J Radiat Oncol Biol Phys* **7**: 497–502

Hilmas DE and Gillette EL (1975) Tumor microvasculature following fractionated x irradiation. *Radiology* **116**: 165–169

Jain RK (1987) Transport of molecules across tumor vasculature. *Cancer Metab Rev* **6**: 559–593

Johansson M, Bergenheim AT, Henriksson R, Koskinen LO, Vallbo C and Widmark A (1997) Tumor blood flow and the cytotoxic effects of estramustine and its constituents in a rat glioma model. *Neurosurgery* **41**: 237–243; discussion 243–244

Johansson M, Bergenheim AT, D'Argy R, Edman K, Gunnarsson P, Widmark A and Henriksson R (1998) Distribution of estramustine in the BT4C rat glioma model. *Cancer Chemother Pharmacol* **41**: 317–325

Kelly PJ, Daumas-Dauport C, Kispert DB, Kall BA, Scheithauer BW and Illig JJ (1987) Imaging based stereotaxic serial biopsies in untreated intracranial glial neoplasms. *J Neurosurg* **66**: 865–874

Leon SP, Folkerth RD and Black PM (1996) Microvessel density is a prognostic indicator for patients with astroglial brain tumors. *Cancer* **77**: 362–372

Lindgren M, Johansson M, Sandstrom J, Jonsson Y, Bergenheim AT and Henriksson R (1997) VEGF and tPA co-expressed in malignant glioma. *Acta Oncol* **36**: 615–618

Littbrand B and Révész L (1969) The effect of oxygen on cellular survival and recovery after radiation. *Br J Radiol* **42**: 914–924

Morales DE, McGowan KA, Grant DS, Maheshwari S, Bhartiya D, Cid MC, Kleinman HK and Schnaper HW (1995) Estrogen promotes angiogenic activity in human umbilical vein endothelial cells in vitro and in a murine model. *Circulation* **91**: 755–763

Nakamura J, Savinov A, Lu Q and Brodie A (1996) Estrogen regulates vascular endothelial growth/permeability factor expression in 7,12-dimethylbenz(a)anthracene-induced rat mammary tumors. *Endocrinology* **137**: 5589–5596

Plate KH, Breier G, Weich HA and Risau W (1992) Vascular endothelial growth factor is a potential tumour angiogenesis factor in human gliomas in vivo. *Nature* **359**: 845–848

Plate KH, Breier G, Millauer B, Ullrich A and Risau W (1993) Up-regulation of vascular endothelial growth factor and its cognate receptors in a rat glioma model of tumor angiogenesis. *Cancer Res* **53**: 5822

Prionas SD, Kowalski J, Fajardo LF, Kaplan I, Kwan HH and Allison AC (1990) Effects of X irradiation on angiogenesis. *Radiat Res* **124**: 43–49

Rubin P and Casarett G (1966) Microcirculation of tumors. II. The supervascularized state of irradiated regressing tumors. *Clin Radiol* **17**: 346–355

Seiler RW, Zimmermann A, Bleher EA and Markwalder H (1979) Preoperative radiotherapy and chemotherapy in hypervascular, high-grade supratentorial astrocytomas. *Surg Neurol* **12**: 131–133

Shweiki D, Itin A, Soffer D and Keshet E (1992) Vascular endothelial growth factor induced by hypoxia may mediate hypoxia-initiated angiogenesis. *Nature* **359**: 843–845

Solesvik OV, Rofstad EK and Brustad T (1984) Vascular changes in a human malignant melanoma xenograft following single-dose irradiation. *Radiat Res* **98**: 115–128

Song CW, Sung JH, Clement JJ and Levitt SH (1974) Vascular changes in neuroblastoma of mice following x-irradiation. *Cancer Res* **34**: 2344–2350

von Schoultz E, Lundblad D, Bergh J, Grankvist K and Henriksson R (1988) Estramustine binding protein and anti-proliferative effect of estramustine in human glioma cell lines. *Br J Cancer* **58**: 326–329

Weidner N (1993) Tumor angiogenesis: review of current applications in tumor prognostication. *Semin Diagn Pathol* **10**: 302–313

Yoshida D, Piepmeier J and Weinstein M (1994). Estramustine sensitizes human glioblastoma cells to irradiation. *Cancer Res* **54**: 1415–1417

Zywietz F (1990) Vascular and cellular damage in a murine tumour during fractionated treatment with radiation and hyperthermia. *Strahlenther Onkol* **166**: 493–501

© Cancer Research Campaign 1999
